# Rapamycin reverses the senescent phenotype and improves immuno-regulation of mesenchymal stem cells from MRL/lpr mice and systemic lupus erythematosus patients through inhibition of the mTOR signaling pathway

**DOI:** 10.18632/aging.100925

**Published:** 2016-04-02

**Authors:** Zhifeng Gu, Wei Tan, Juan Ji, Guijian Feng, Yan Meng, Zhanyun Da, Genkai Guo, Yunfei Xia, Xinhang Zhu, Guixiu Shi, Chun Cheng

**Affiliations:** ^1^ Department of Rheumatology, Affiliated Hospital of Nantong University, Nantong, Jiangsu Province 226001, China; ^2^ Department of Emergency Medicine, The Yangzhou First People's Hospital, Yangzhou, Jiangsu Province 225001, China; ^3^ Department of Stomatology, Affiliated Hospital of Nantong University, Nantong, Jiangsu Province 226001, China; ^4^ Department of Rheumatology, Affiliated First Hospital of Xiamen University, Xiamen, Fujian Province 361000, China; ^5^ Jiangsu Province Key Laboratory for Inflammation and Molecular Drug Target, Medical College of Nantong University, Nantong, Jiangsu Province 226001, China

**Keywords:** rapamycin (RAPA), mesenchymal stem cells (MSCs), systemic lupus erythematosus (SLE), senescence, immunoregulation

## Abstract

We have shown that bone marrow (BM)-derived mesenchymal stem cells (BM-MSCs) from SLE patients exhibit senescent behavior and are involved in the pathogenesis of SLE. The aim of this study was to investigate the effects of rapamycin (RAPA) on the senescences and immunoregulatory ability of MSCs of MRL/lpr mice and SLE patients and the underlying mechanisms. Cell morphology, senescence associated β-galactosidase (SA-β-gal) staining, F-actin staining were used to detect the senescence of cells. BM-MSCs and purified CD4+ T cells were co-cultured indirectly. Flow cytometry was used to inspect the proportion of regulatory T (Treg) /T helper type 17 (Th17). We used small interfering RNA (siRNA) to interfere the expression of mTOR, and detect the effects by RT-PCR, WB and immunofluorescence. Finally, 1×10^6^ of SLE BM-MSCs treated with RAPA were transplanted to cure the 8 MRL/lpr mice aged 16 weeks for 12 weeks. We demonstrated that RAPA alleviated the clinical symptoms of lupus nephritis and prolonged survival in MRL/lpr mice. RAPA reversed the senescent phenotype and improved immunoregulation of MSCs from MRL/lpr mice and SLE patients through inhibition of the mTOR signaling pathway. Marked therapeutic effects were observed in MRL/lpr mice following transplantation of BM-MSCs from SLE patients pretreated with RAPA.

## INTRODUCTION

Systemic lupus erythematosus (SLE) is a chronic autoimmune inflammatory disease characterized by multi-organ involvement and a remarkable variability in clinical presentation [1]. It is a typical autoimmune disease based on the variety of its proposed pathogenesis, including abnormalities of T and B lymphocytes. Current treatments of severe SLE flares consist of toxic immunosuppressive drugs, most commonly cyclophosphamide, mycophenolate mofetil, and leflunomide [2]. However, the therapeutic options in SLE patients who are refractory to standard treatments are extremely limited, and the disease remains potentially fatal in some patients[3]. With recent advances in our understanding of the underlying pathology, several new strategies have been developed to target specific activation pathways relevant to the pathogenesis of SLE [4, 5]. For example, B-cell-depleting therapies using the monoclonal antibody rituximab and the B lymphocyte stimulator (BLyS) inhibitor belimumab, have been shown be beneficial in a specific subpopulation of lupus patients [6, 7].

Mesenchymal stem cells (MSCs) are widely identified as a promising cell source because of their clonogenic, self-renewal and pluripotent differentiation ability [8]. MSCs have been found to possess immunomodulatory effects on various activated immune cells, such as T cells, B cells, natural killer cells and dendritic cells [9-11]. Additionally, MSCs are able to escape alloantigen recognition because of their low immunogenicity and accompanying lack of expression of costimulatory molecules [12, 13]. These properties make MSCs promising candidate cells for preventing rejection in organ transplantation and in the treatment of autoimmune disease.

Our studies and those of others have revealed that SLE BM-MSCs exhibit slow growth with early signs of senescence, as well as some immunoregulatory abnormalities [14-17]. Accumulating evidence confirms the safety and efficacy of allogeneic MSC transplantation in treating drug-resistant SLE patients and animal models [18-24]. However, Carrion and coworkers reported that autologous BM-MSC transplantation (MSCT) had no effect on disease activity in two SLE patients [25]. These findings suggested that the senescence of MSCs from SLE patients may contribute to the disease pathogenesis. Therefore, a complete understanding of the mechanisms underlying early senescence of MSCs in SLE patients is required.

Mammalian target of rapamycin (mTOR) integrates nutrient and hormonal signals to function as a central regulator of diverse cellular processes including cell growth [26-29]. It is a phosphatidylinositol kinase-related kinase (phosphatidylinositol kinase-related kinase, PIKK) protein family member, and regulates protein translation and cell growth and proliferation via phosphorylation of the downstream target proteins p70 ribosomal protein S6 kinase (p70S6K) and eukaryotic initiation factor 4E binding protein1 (4EBP1) [30, 31]. Previous studies revealed that the mTOR signaling pathway is involved in a variety of biological processes including cell senescence *in vivo* [32-36]. Rapamycin (RAPA), which is an inhibitor of the mTOR signaling pathway, is a macrolide antibiotic with potent immunosuppressive properties [37, 38]. Recent studies have shown that RAPA can decelerate certain aspects of cellular senescence [39-42]. In addition, the therapeutic use of RAPA in SLE patients and animal models is clinically effective. RAPA has been shown to normalize T cell activation-induced calcium flux in patients with SLE [43]. However, the ability of RAPA to alleviate LN by influencing the senescence of BM-MSCs from SLE patients and the therapeutic potential of MSCs *in vitro* autotransplantation have not yet been reported.

In this study, we further confirmed that RAPA alleviates LN and prolongs the survival of MRL/lpr mice. Interestingly, we have found that RAPA reversed the senescent phenotype and improved the immuno-regulatory ability of MSCs from MRL/lpr mice. Furthermore, we report, for the first time, the involvement of the activated mTOR pathway in the senescence of MSCs from SLE patients and demonstrated marked therapeutic effects of MRL/lpr mice following transplantation of RAPA-pretreated BM-MSC obtained from SLE patients.

## RESULTS

### RAPA improves lupus nephritis by influencing cellular senescence in BM-MSCs from MRL/lpr mice

**Figure 1:**
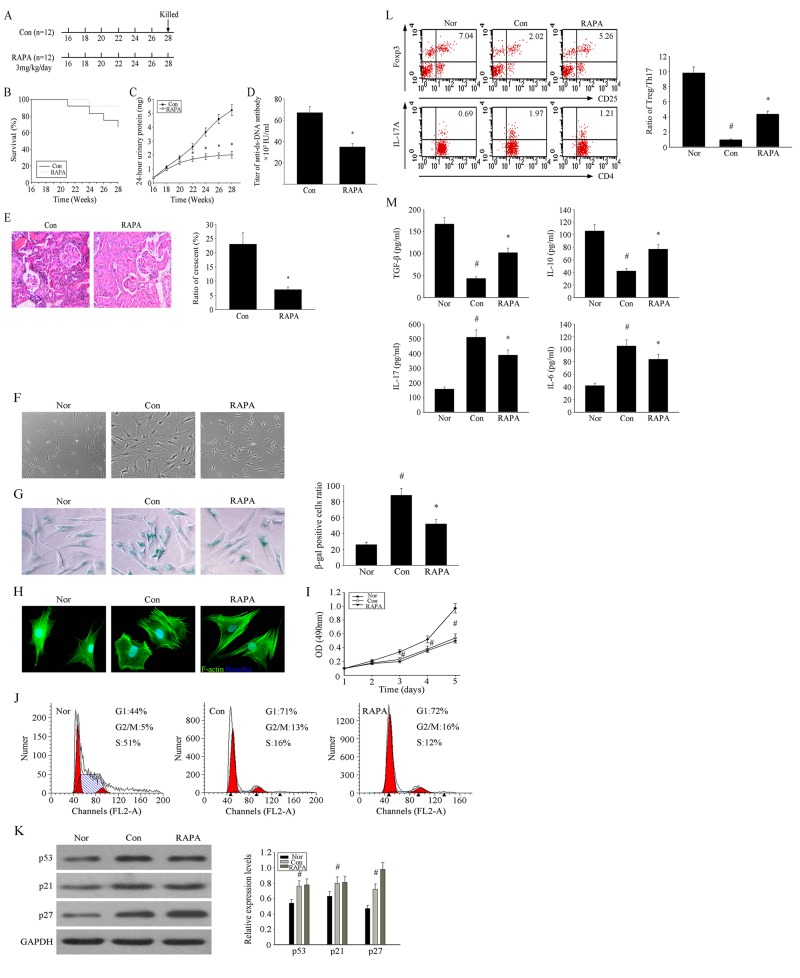
RAPA improves lupus nephritis by influencing the cellular senescence of BM-MSCs from MRL/lpr mice (**A**) The treatment group had intragastric administration of RAPA 3mg/kg/day between 16 and 28 weeks of ages. Survival curves observed that The survival rate of the RAPA-treated MRL/lpr mice was higher than that of control group. (**B**-**E**) We recorded the survival rate and the weight of mice. 24-hours urinary protein was measured by coomassie brilliant blue method. Mice were killed and were taken peripheral blood in orbit. Elisa showed that anti-ds-DNA antibody titer in serum in RAPA -treated group is lower than control group. HE-staining showed that renal pathological changes of control group is significant,including glomerular sclerosis, mesangial cell proliferation,matrix widened, and formation of crescent, a number of lymphocytes infiltrating the interstitium (HE×300). However, histopathological changes of RAPA -treated group were remarkable alleviated. (**F**) Cellular morphology observed that the RAPA-treated MSCs from MRL/lpr mice were less hypertrophic than untreated group (magnification; ×200). (**G**) MSCs were fixed and stained for β-gal. The number of SA-β-gal- positive cells among treated groups decreased in comparison with untreated group. (**H**) Immunofluorescence showed that the abnormal F-actin distribution in MSCs from MRL/lpr mice was reversed by RAPA treatment. (**I**) Cell Counting Kit (CCK)-8 method was used to detect the cell proliferation rate. (**J**) Flow cytometry was used to detect the distribution of cell cycle. (**K**) Western Blotting was used to detect the changes of cell cycle-related proteins. However, no remarkably differences were found between treated and untreated MSCs. (**L**) P4 MSCs transwell cultured with CD4+ T cells for 72 h. The count of Treg cells decreased and Th17 cells increased in MSCs from MRL/ lpr mice compared to the normal group by flow cytometry analysis. MSCs from RAPA-treated group could reverse the abnormal changes. The statistical results revealed that the treatment of RAPA could up-regulated the ratio of Treg/Th17 from MRL/lpr mice MSCs. (**M**) The supernatants of MSCs were collected. RAPA-treated group induced the secretion of IL-10 and TGF-β but reduced IL-17 and IL-6 by ELISA. All data were expressed as the mean±SEM (n = 3, *P<0.05 compared with normal group, #P<0.05 compared with the untreated group).

Previous studies have demonstrated the clinical efficacy of RAPA for the treatment of SLE patients and in animal models of lupus. The scheme of RAPA treatment procedures used in the present study is shown in Figure 1A. RAPA increased the survival rate of MRL/lpr mice (Fig. 1B) and alleviated symptoms of LN, including 24-h urinary protein, serum anti-ds-DNA antibody levels, and glomerular sclerosis (Fig. 1C–E). MSCs from MRL/lpr mice showed senescent behavior, characterized by flattened and enlarged cell morphology, increased SA-β-gal activity, and disordered cytoskeletal distribution. Interestingly, we observed decelerated cell hypertrophy in BM-MSCs in the RAPA-treated group (Fig. 1F) and the number of SA-β-gal-positive cells (Fig. 1G). The disordered distribution of F-actin was also reversed by RAPA treatment (Fig. 1H). In contrast, proliferation of BM-MSCs was not affected by RAPA treatment (Fig. 1I–K).

### Previous studies have shown abnormalities in the immunoregulatory ability of that MSCs from MRL/lpr mice

In the present study, we examined the influence of BM-MSCs on the production of Treg and Th17 cells. BM-MSCs from MRL/lpr mice were cultured in transwells with BALB/c splenic CD4+T cells for 72 h. We found that RAPA-treated MSCs from MRL/lpr mice upregulated the number of Treg cells and down-regulated the number of Th17 cells to increase the ratio of Treg/Th17 (Fig. 1L). At the same time, RAPA treatment increased the secretion of regulatory cytokines TGF-β and IL-10, but decreased that of the proinflammatory cytokines IL-17 and IL-6 in these cultures (Fig. 1M). These results implied that RAPA treatment decelerated the senescence of BM-MSCs from MRL/lpr mice but had no effect on cell cycle arrest and promoted the immunoregulatory ability of MSCs from MRL/lpr mice by enhancing the ratio of Treg/Th17 cells and influencing the profile of related cytokine secretion. Reversing MSC senescence may be an effective approach to SLE therapy.

### RAPA inhibited the overactivation mTOR pathway to reverse the senescence of BM-MSCs from MRL/lpr mice

**Figure 2:**
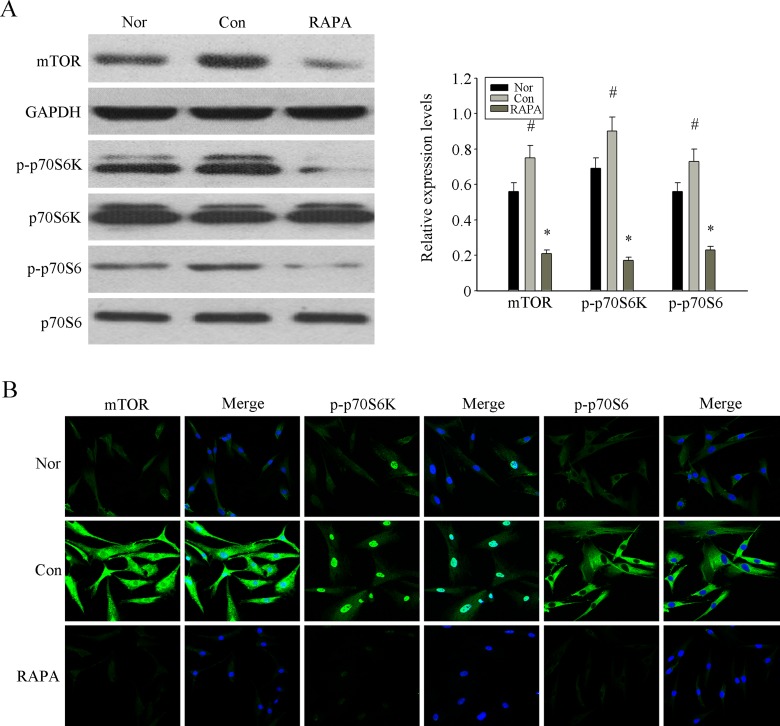
RAPA inhibited the over-activation mTOR pathway to revers the senescence of MSCs from MRL/lpr mice (**A**) The over-expression of p-mTOR, p-S6K and p-S6 in p4 MSCs from MRL/lpr mice compared with normal group were determined by western blot analysis. The treatment of RAPA could obviously inhibit the expression of those proteins. GAPDH was used as the internal control. (**B**) P4 MSCs cultured into 24-well plates. The expression of p-mTOR, p-S6K and p-S6 analyzed by immunofluorescence staining showed that their over-activation in MSCs from MRL/lpr mice could be inhibited by RAPA treatment. Counterstaining with DAPI displays the localization of the nucleus (Scale bar = 50 μm). All data were expressed as the mean±SEM (n = 3,*P<0.05 compared with normal group, #P<0.05 compared with the untreated group).

Previous studies have shown that the mTOR signaling pathway is a central mechanism of cellular senescence [26-28]. Activated mTOR phosphorylates S6K, which in turn phosphorylates S6 [30, 31]. Therefore, we investigated the expression of p-mTOR, p-S6K and p-S6 in MSCs from MRL/lpr mice, normal group and RAPA-treated group by Western blot analysis. We found higher levels of phosphorylated mTOR, S6K and S6 in MSCs from MRL/lpr mice compared to the normal group; this difference was reversed in the RAPA-treated group (Fig. 2A). Similarly, immunofluorescence analysis showed that RAPA reversed the high intracellular expression of p-mTOR, p-S6K and p-S6 in MRL/lpr mice MSCs (Fig. 2B). These results confirmed that RAPA played an inhibitory role in the mTOR pathway of MSCs from MRL/lpr mice.

### Overactivation of the mTOR pathway is involved in the senescence of MSCs from SLE patients

**Figure 3:**
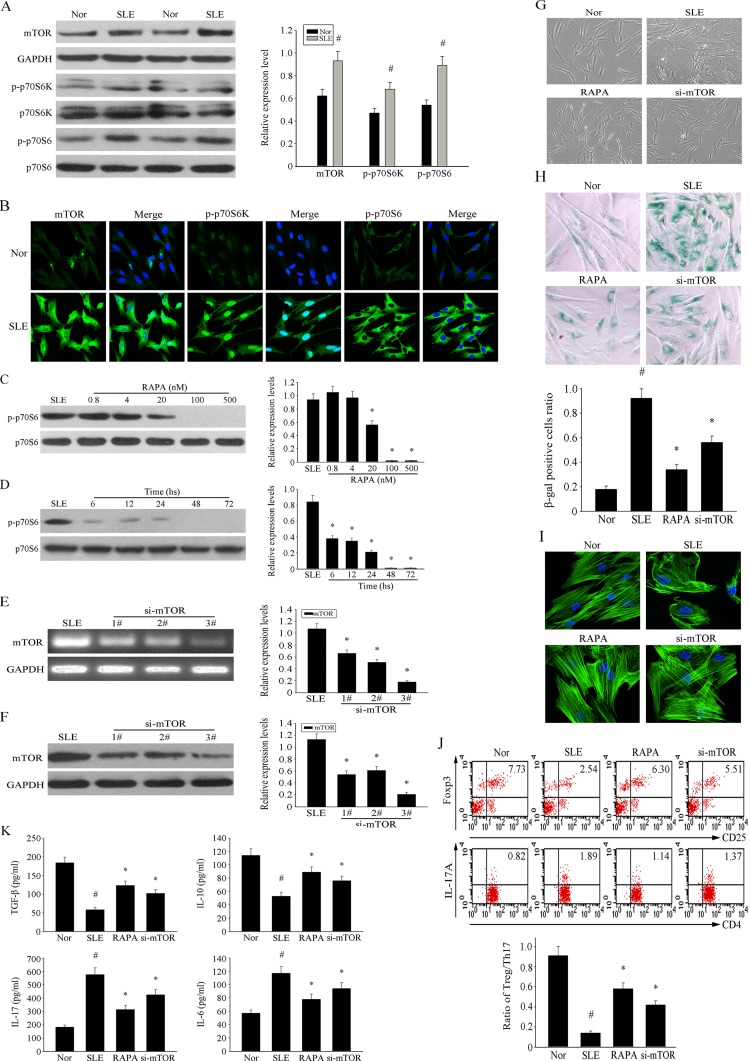
Over-activation of mTOR pathway was involved in the senescence of MSCs from SLE patients (**A**) The over-expression of p-mTOR, p-S6K and p-S6 in MSCs from SLE compared with normal group were determined by western blot analysis. GAPDH was used as the internal control. (**B**) P4 MSCs from SLE patients and normal group were cultured in 24-well plates. Immunofluorescence staining of p-mTOR, p-S6K and p-S6 verified their over-activation in SLE MSCs. Counterstaining with DAPI displays the localization of the nucleus (Scale bar = 50 μm). (**C**-**D**) P4 MSCs from SLE patients cultured at the different concentration of RAPA for 72 h. RAPA achieved maximal effects at about 500 nM by assaying the inhibition of p-70S6. (E-F) BMMSCs were depleted of mTOR by RNAi. The third one was chosen as the best siRNA by western blotting. (**G**) P4 MSCs from SLE patients were treated with 500 nM RAPA and si-mTOR or not for 72 h. Cellular morphology showed that the RAPA and si-mTOR treated SLE MSCs were less hypertrophic than untreated group (magnification; ×200). (**H**) MSCs were fixed and stained for β-gal. The number of SA-β-gal- positive cells obviously decreased among treated SLE MSCs in comparison with untreated group. (**I**) Immunofluorescence showed that RAPA and si-mTOR reversed abnormal F-actin distribution in MSCs from SLE patients. (**J**) P4 SLE MSCs were treated with 500 nM RAPA and the third si-mTOR or not, then transwell cultured with CD4+T cells for 72 h. The count of Treg cells decreased and Th17 cells increased in SLE MSCs compared to the normal group by flow cytometry analysis. But si-mTOR and RAPA-treated MSCs could reverse the abnormal changes. The statistical results revealed that RAPA could up-regulated the ratio of Treg/Th17 from SLE MSCs, which was down-regulated compared with normal group. (**K**) The supernatants of MSCs were collected. si-mTOR RAPA-treated SLE MSCs induced the secretion of IL-10 and TGF-β but reduced IL-17 and IL-6 by ELISA. All data were expressed as the mean±SEM (n = 3, *P<0.05 compared with normal group, #P<0.05 compared with the untreated group).

To further confirm its role in the senescence of MSCs from SLE patients, we examined the expression of components of the mTOR pathway. As shown in Figure 3A, the phosphorylation levels of mTOR and its proteins expressed by its downstream regulated genes were higher in SLE MSCs compared to the normal group. Furthermore, immunofluorescence analysis confirmed higher expression of intracellular p-mTOR, p-S6K and p-S6 levels in SLE MSCs compared with the normal group (Fig. 3B). To confirm the role of the mTOR pathway in the senescence of MSCs in SLE patients, we investigated the dose- and time-dependent inhibitory effects of RAPA on the mTOR pathway by measuring S6 phosphorylation, which is a significant marker of mTOR activity. Results showed that RAPA inhibited S6 phosphorylation at concentrations 20 nM or higher, achieving maximal effects at 100 nM–500 nM (Fig. 3C). Furthermore, 500 nM RAPA achieved maximal effects at 72 h (Fig. 3D). Thus, all subsequent experiments were performed using RAPA at 500 nM for 72 h; S6 phosphorylation was completely inhibited under these conditions.

To further determine the effects of the mTOR signaling pathway on senescence and the immunoregulatory ability of MSC, we depleted MSC of mTOR by RNAi treatment. As shown in Figures 3E–F, by comparing interference effects of three RNAi sequences, we chose the third one to do the following experiments. We found that both RNAi and RAPA treatment reversed the senescent behavior of MSCs from SLE patients. The MSCs from SLE patients were larger than those in the normal group, and exhibited more numerous and longer podia. Morphological evaluation showed that RAPA and siRNA decelerated the hypertrophy of MSCs from SLE patients (Fig. 3G). It is noteworthy that, compared with normal MSCs, RAPA was less effective at preventing hypertrophy than siRNA, indicating that mTOR-dependent and independent components influence cell size and growth. SA-β-gal was usually used to examine cellular senescence. The number of SA-β-gal-positive cells was notably increased in SLE MSCs and this number was decreased by RAPA and knockdown of mTOR (Fig. 3H). Immunofluorescence analysis showed that the F-actin distribution was disorderly and assembled around the nuclear region in MSCs from SLE patients. This abnormal distribution of F-actin was effectively reversed by RAPA and si-mTOR treatment (Fig. 3I); however, the proliferation rate of MSCs was not affected (data not shown).

We investigated the immunoregulatory ability of SLE MSCs on CD4+T cells using a transwell culture system. We found that RAPA treatment and knockdown of mTOR increased the number of Treg cells and decreased the number of Th17 cells generated in transwell cultures; thus, increasing the Treg/Th17 ratio. Our data revealed that RAPA increased the ratio of Treg/Th17 generated in the presence of SLE MSCs, but was lower than that in the normal control group (Fig. 3J). Furthermore, both treatments increased the secretion of regulatory cytokines TGF-β and IL-10, but reduced the levels of the proinflammatory cytokines IL-17 and IL-6 in MSCs from SLE patients (Fig. 3K). Taken together, these results demonstrated the involvement of the mTOR pathway in the senescence of MSCs from SLE patients. RAPA decelerated the senescence of MSCs from SLE patients and increased the Treg/Th17 cell ratio stimulated by MSCs from SLE patients by affecting the secretion of cytokines.

### MSCs from SLE patients pretreated with RAPA have a significant therapeutic effect on LN of MRL/lpr mice

**Figure 4:**
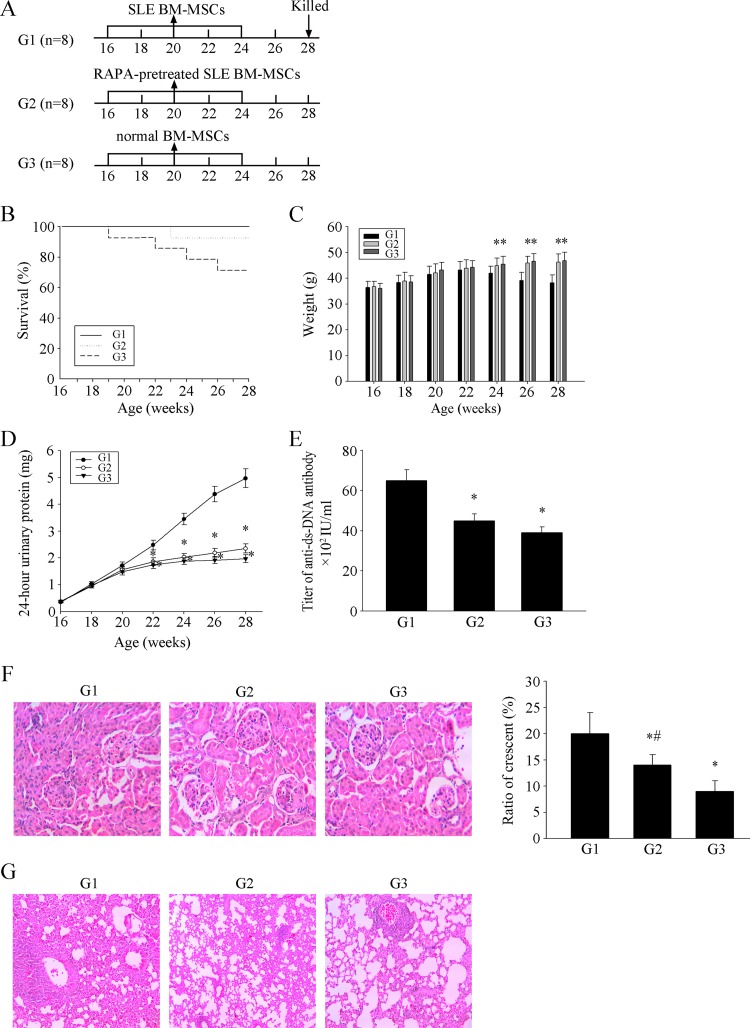
MSCs from SLE patients pretreated with RAPA have a significant effect on LN of MRL/lpr mice (**A**) 24 MRL / lpr mice were divided into three groups: SLE BM-MSCs transplantation group (G1), RAPA-pretreated SLE BM-MSCs transplantation group (G2), normal BM-MSCs transplantation group (G3). Three groups were injected with BM-MSC in tail vein. (**B**) Survival curves observed that The survival rate of G2 and G3 MRL/lpr mice was higher than that of control group. (**C**) Three groups MRL/lpr mice were weighed one time two weeks. Weight between G2, G3 and G1 had obvious difference from 24 weeks. (**D**) 24-hours urinary protein was measured by coomassie brilliant blue method. (**E**) mice were killed and were taken peripheral blood in orbit. Elisa showed that anti-ds-DNA antibody titer in serum in G2 and G3 is lower than G1. (**F**) HE-staining showed that renal pathological changes of G1 is significant, including glomerular sclerosis, mesangial cell proliferation, matrix widened, and formation of crescent, a number of lymphocytes infiltrating the interstitium (HE×300). However, histopathological changes of other groups were remarkable alleviated. (**G**) HE-staining showed that pulmonary pathological changes of G1 is significant, including Pulmonary vascular congestion and edema, lymphocyte and mononuclear cell infiltration, and consolidation of lung (HE×300). However, histopathological changes of other groups were remarkable alleviated. There is not statistical difference between G2 and G3. All data were expressed as the mean±SEM (n = 3, *P<0.05 compared with normal group, #P<0.05 compared with the untreated group).

Recent studies have indicated that allogenic MSCT is a feasible and safe therapeutic strategy in lupus-prone mice and SLE patients[18-24]. However, syngeneic BM-MSCT was ineffective [22]. Therefore, we investigated this issue in transplantation experiments conducted in MRL/lpr mice (Fig. 4A). As shown in Figure 4B, the survival rates in the RAPA-pretreated SLE MSCs transplantation group (G2) and normal MSCs transplantation group (G3) were higher than that in the SLE MSCs transplantation group (G1). The weight of the mice in G2 and G3 gradually increased (Fig. 4C). The 24-hours urinary protein (Fig. 4D) and serum anti-ds-DNA antibody levels in G2 and G3 were lower than those in G1 (Fig. 4E). In terms of pathology, glomerular sclerosis and interstitial fibrosis (Fig. 4F) and pulmonary inflammation (Fig. 4G) were ameliorated in G2 and G3 MRL/lpr mice. These results demonstrated that transplantation of MSCs from SLE patients pretreated with RAPA and normal MSCs have a significant therapeutic effect on LN in MRL/lpr mice.

## DISCUSSION

Since 1994, when the significant reduction or prevention of the many pathologic features of lupus normally seen in the MRL/l mouse mediated by RAPA were first reported [44], there have been an increasing number of studies focusing on RAPA treatment of lupus. In 2006, Fernandez et al. reported that RAPA appeared to be a safe and effective therapy for SLE in patients who proved refractory to traditional medications. This group showed that RAPA normalized T cell activation-induced calcium flux in patients with SLE[43]. In the study, we confirmed that RAPA attenuated the severity of established nephritis. Furthermore, we demonstrated for the first time that RAPA reversed the senescent phenotype and improved the regulatory ability of MSCs from MRL/lpr mice and SLE patients by inhibition of the mTOR signaling pathway. Therefore, reversal of the senescence of MSC may be an effective approach to SLE therapy.

The mTOR signaling pathway plays an important role in cellular senescence [26-29]. Yentrapalli and others have shown that the PI3K/AKT/mTOR signaling pathway is involved in senescence of primary human endothelial cells induced by long-term radiation [35]. In addition, Gharibi reported that the AKT/mTOR signaling pathway promoted MSC senescence and reduced osteogenic differentiation [36]. Our study shows that the mTOR signaling pathway of MSCs from SLE patients is considerably more active than that of the normal group as demonstrated by the upregulated phosphorylation of mTOR, S6K and S6. Furthermore, siRNA-mediated mTOR knockdown inhibited the senescence of MSCs and restored their immunomodulatory capacity. This evidence suggests that the mTOR pathway is involved in the accelerated senescence of MSCs in SLE patients.

Emerging evidence indicates the potential of RAPA to alleviate cellular senescence [45, 46]. Chen et al. confirmed that RAPA-mediated suppression of the mTORC1 prevented the appearance of senescence markers in retinal pigment epithelium [41]. Cao et al. showed that RAPA abolished nuclear blebbing, delayed the onset of cellular senescence, and enhanced the degradation of progerin (an abnormal form of the lamin A protein) in Hutchinson-Gilford Progeria Syndrome (HGPS) cells [39]. In our study, we found that RAPA decelerated senescence of SLE BM-MSCs by inhibiting excessive cellular growth caused by the mTOR pathway. Furthermore, the cellular morphology became progressively less hypertrophic in comparison with untreated SLE BM-MSCs, which exhibited more numerous and extensive podia, which contained more actin stress fibers. In addition, fewer SA-β-gal-positive cells were detected among RAPA-treated SLE BM-MSCs, while the F-actin was distributed in a disordered pattern and assembled mainly in the nuclear region. Remarkably, we also demonstrated that RAPA did not stimulate the proliferation of BM-MSCs from SLE, did not abrogate cell cycle arrest caused by p21^Cip1^ and p27, and did not force cells to bypass cell cycle arrest.

More importantly, we found that after transwell culture CD4+ T cells with MSCs for 72 h, the ratio of Treg/Th17 generated in the presence of the RAPA and si-mTOR treated SLE MSCs was increased compared to those cultured in the presence of the untreated SLE MSCs. Defects in Treg and/or Th17 development, maintenance or function have been found to be associated with several human autoimmune diseases[47-49]. Moreover, Sun et al. demonstrated that MSCs from NZBW/F1 lupus mice and SLE human patients had defective immunoregulatory function compared with healthy controls [50-52]. It has been reported that SLE flares might be linked to the expansion of the Th17 cell population and the depletion of the natural Treg cell subpopulations. In contrast to the role of Th17 cells, Treg cells play an essential role in maintaining immune homeostasis and preventing autoimmunity [53]. TGF-β and IL-10 are critical differentiation factor for the generation of Treg cells [54], while IL-6 and IL-17 have been shown to be the main factors responsible for the reciprocal regulating proinflammatory Th17 cell development[55]. Our results showed that RAPA and si-mTOR treated SLE MSCs induced the secretion of IL-10 and TGF-β, but downregulated IL-17 and IL-6. Thus, our data demonstrates that RAPA improves the immuno-regulatory capacity of MSCs from SLE patients and indicates the involvement of the mTOR signaling pathway in the immune disorders of SLE patients.

Although syngeneic BM-MSCT has proved ineffective, previous reports have shown the clinical efficacy of allogeneic MSCT in SLE[18-24]. However, several barriers to the application of allogeneic transplantation exist, such as ethical considerations, the scarcity of donors and the risk of contamination. In this study, we showed that transplantation RAPA-pretreated SLE MSCs into MRL/lpr mice alleviated LN and improved the survival rate in recipients. These results suggest that RAPA reverses the senescence of MSCs in SLE patients.

In conclusion, we have demonstrated for the first time that mTOR plays a key role in senescence and immune disorders of MSC from SLE patients and MRL/lpr mice. Furthermore, we show that RAPA reverses the senescence and immune disorders by mTOR signaling pathway. Moreover, these observations indicate the therapeutic potential of autologous MSCT following *in vitro* intervention to treat SLE. Our findings indicate that targeting mTOR in MSCs may provide a new therapeutic strategy for the treatment of SLE patients.

## MATERIALS AND METHODS

### Patients

Twelve SLE patients aged 15 to 38 years (mean 25.43±5.75 years) were enrolled in the study. The SLE diagnosis was made based on the criteria proposed by the American College of Rheumatology. Twelve healthy subjects (all female), with a similar age distribution (mean 24.86±4.22 years) were enrolled as the normal control group. All individuals gave informed consent to participation in the study, which was approved by the Ethics Committee of the Affiliated Hospital of Nantong University (China).

### Isolation, culture and identification of BM-MSCs from SLE patients and normal subjects

MSCs were isolated and expanded from the iliac crest BM of all SLE patients and normal subjects. Five milliliters of heparinized BM were mixed with an equal volume of phosphate-buffered saline (PBS). The resuspended cells were then layered over Ficoll solution (1.077 g/mL) and centrifuged at 2,000 ×*g* for 25 min at room temperature. The mononuclear cells were collected from the interface and resuspended in low-glucose Dulbecco Modified Eagle Medium (L-DMEM) supplemented with 10% heat inactivated fetal bovine serum (FBS). The cells were then plated at a density of 2×10^7^ cells per 25 cm^2^ dish and cultured at 37°C in a 5% CO_2_ incubator. After 5 days, the medium was replaced and non-adherent cells were removed; this process was repeated every three days thereafter. When the MSCs became nearly confluent, the adherent cells were released from the dishes with 0.25% trypsin-EDTA (Gibco, USA), and then replated at a density of 1×10^6^ cells per 25 cm^2^ dish. Flow cytometric analysis showed that the cells were positive for CD29, CD44, CD105, and CD166, but negative for CD14, CD34, CD38, CD45 and HLA-DR (data not shown). After passage 4 (p4), cells were used for the following studies.

### Mice and treatments

Eight-week-old female MRL/lpr mice (n = 48) and BALB/c mice (n = 14) (Slyke Experimental Animals Company, China) were divided into five groups. One group of MRL/lpr mice (n = 12) was treated with RAPA (3 mg/kg/day, Sigma-Aldrich, USA) by oral gavage from 16 to 28 weeks. Another group (n = 12) of control MRL/lpr mice received an equal volume of normal saline using the same schedule. The remaining MRL/lpr mice were randomly divided into the following three groups: Group 1 (G1, n = 8) were transplanted with BM-MSCs from SLE patients; Group 2 (G2, n = 8) were transplanted with 500 nM RAPA-pretreated BM-MSCs from SLE patients; Group 3 (G3, n = 8) were transplanted with normal BM-MSCs. The experimental protocols conformed to the animal care guidelines of the China Physiologic Society and were approved by our Institutional Animal Research Committee.

### Albuminuria

24-h urine samples were collected from each mouse by metabolic cages method once every two weeks. Urinary albumin levels were measured using a commercially available ELISA kit (BioAssay Systems, Hayward, CA, USA) according to the manufacturer's instructions.

### Anti-dsDNA antibody measurements

Blood was collected from the mice by cardiac puncture under anesthesia at the time of euthanization. Serum levels of anti-dsDNA (IgG) antibody were determined using a commercially available ELISA kit (Alpha Diagnostic International, San Antonio, TX, USA) according to the manufacturer's instructions.

### Renal and pulmonary histology studies

At the time of euthanization, kidney and lung specimens were obtained, fixed in 10% formaldehyde and embedded in paraffin. Sections (4 μm thickness) were prepared and then stained with haematoxylin and eosin (H&E). The kidney and lung sections were coded and examined by two independent observers who were blinded to the treatment groups. At least 50 glomeruli were examined for each sample.

### Isolation, culture and identification of BM-MSCs from MRL/lpr mice

The BM was flushed out of the femurs and tibias removed from MRL/lpr and BALB/c mice using 10 ml PBS with 100 U/ml heparin in a syringe. The cells were centrifuged at 1000 ×*g* for 10 min. The cell pellet was resuspended in 5 ml L-DMEM supplemented with 10% FBS (Gibco, USA) and plated in a 25 cm^2^ dish to allow the MSCs to adhere. Cultures were maintained in a humidified atmosphere with 5% CO_2_ at 37°C. Flow cytometric analysis showed that the cells were positive for CD29, CD44, CD105, and CD166, but negative for CD14, CD34, CD38, CD45 and HLA-DR (data not shown). MSCs were cultured using the same method as that used for human MSCs. All experiments were conducted with MSCs at p4.

### Western blotting

BM-MSCs were washed in cold-buffered PBS and were then lysed in RIPA buffer (150 mM NaCl, 1%TritonX-100, 0.5%NaDOD, 0.1%SDS and 50 mM Tris, pH 8.0). After centrifugation (12,000 rpm, 5 min) at 4°C, the protein supernate was transferred into new tubes. The protein concentration of the samples was determined with a bicinchoninic acid protein assay (Pierce, USA). Equal amounts of proteins were separated by 10% SDS polyacrylamide gel electrophoresis (PAGE) and electrophoretically transferred to polyvinylidene difluoride (PDVF) membranes. Membranes were blocked with 5% non-fat milk in TBST (20 mM Tris, 150 mM NaCl, 0.05% Tween-20) and incubated with primary antibodies (1:500) at 4°C overnight. Subsequently, the membranes were incubated with horseradish peroxidase conjugated mouse anti-rabbit secondary antibody for 2 h at room temperature. The blots were developed using an enhanced chemiluminescence kit (NEN Life Science Products, Boston, MA, USA). GAPDH was used as a reference protein. The following primary antibodies were used: GAPDH (anti-rabbit, Santa Cruz), p-mTOR (anti-rabbit, Sigma), mTOR (anti-rabbit, Sigma), p-S6K (anti-rabbit, Santa Cruz), S6K (anti-rabbit, Santa Cruz), p-S6 (anti-rabbit, Sigma), S6 (anti-rabbit, Sigma), p53 (anti-rabbit, Cell Signaling), p21 (anti-rabbit, Sigma), p27 (anti-rabbit, Cell Signaling).

### Immunofluorescence

BM-MSCs were fixed with 4% paraformaldehyde (PFA) for 1 h, washed with PBS containing 0.1% Triton X-100 (PBST), and blocked for 30 min in PBST supplemented with 10% FBS. Cells were then incubated with one of the following primary antibodies (1:100) in the same solution overnight at 4°C: p-mTOR (anti-rabbit, Sigma), p-S6K (anti-rabbit, Santa Cruz), p-S6 (anti-rabbit, Sigma), washed and incubated in the dark with goat anti-rabbit- (cy3-) conjugated antibodies (1 : 300, ICN Cappel, USA) for 2 h at room temperature. Nuclei were stained with DAPI (1:800, Santa Cruz). The cells were examined with a Leica fluorescence microscope (Germany). For visualization of the MSC cytoskeleton, cells were washed twice with PBS and fixed in 4% PFA for 1 h. After permeabilization and blocking, they were then incubated with fluorescein isothiocyanate-conjugated phalloidin, which is a high-affinity filamentous probe. The stained cells were then examined under a Zeiss Confocal Laser Scanning Microscope.

### Senescence-associated-β-galactosidase assay

The SA-β-gal activity was determined using the in situ β-galactosidase staining kit from the Beyotime Institute of Biotechnology following the manufacturer's instructions. MSCs treated with or without RAPA were passaged into 6-well culture plates at a density of 5×10^4^ cells per well for 72 h. The cells were then washed twice with PBS and fixed with the 4% paraformaldehyde for 15 min. After incubation with the SA-β-gal detection solution at 37°C without CO_2_ overnight, the cells were washed and analyzed under the microscope (Leica company, Germany). We counted at a minimum of 500 cells to determine the percentage of SA-β-gal-positive cells.

### Cell number assay

MSCs were seeded at 0.7×10^4^ cells/well in 6-well plates in triplicate for each condition. RAPA (500 nM) was added to the cultures of SLE BM-MSCs, and dimethylsulfoxide (DMSO) was added to the untreated control cells. Cells were collected at 1 to 4 days after plating, dissociated, and the total cell numbers were counted.

### CD4+ T cell isolation and transwell culture with MSCs

Single-cell suspensions of spleens collected from the BALB/c mice were prepared by mechanical disruption in PBS. CD4+ T cells were isolated by magnetic sorting of Dynabead-bound mouse CD4+ cells according to the manufacturer's directions (Dynal Biotech). Positively selected cells contained an average of 99% CD4+ T cells as assessed by flow cytometric analysis with CD4 monoclonal antibody. Cells were cultured in RPMI 1640 medium supplemented with 10% FCS, 1×nonessential amino acids and 1 mM sodium pyruvate. The purified CD4+ T cells (1×10^5^) were obtained and cultured in the lower chamber of the 24-well diameter transwell plate with a 0.3-μm pore size membrane (Corning, NY, USA). MSCs (1×10^6^) were seeded onto the transwell membrane of the inner chamber for 72 hours of transwell culture.

### Flow cytometry

For cell cycle analysis, MSCs treated with or without 500 nM RAPA were collected and fixed with 70% ethanol at 4°C for 24 h. After being washed with PBS and then treated with 100 μg/ml RNase (Sigma, USA) for 30 min, the cells were stained with 50 μg/ml propidium iodide (PI) solution (Sigma, USA) for 30 minutes and analyzed by flow cytometry (FACS Calibur, BD Biosciences, USA). The fraction of cells in the G0/G1, S, and G2/M phases were quantified with the ModFit LT system. Three separate experiments were performed.

For regulatory T (Treg) and T helper type 17 (Th17) cell analysis, the ratio of Treg/Th17 among the CD4+ T cell population was analyzed using Treg and Th17 assay kits (Santa Cruz, USA). After 72 h of transwell culture, CD4+ cells were harvested and washed with PBS, resuspended in 100 μl staining buffer and divided into two aliquots (one for detection and another for the isotype control). The CD4+ T cells were stained with anti-CD25-APC or anti-IL-17-PE mAbs to assay the Treg and Th17 cells, respectively. IgG_2a_-PE rat was used as the isotype control. All procedures were performed according to the manufacturer's protocol and cells were analyzed by flow cytometry (FACS Calibur, BD Biosciences, USA).

### Cytokine determination by ELISA

The concentrations of transforming growth factor (TGF)-β, interleukin (IL)-10, IL-17 and IL-6 cytokines released in the transwell culture supernatants were measured by enzyme-linked immunosorbent assay (ELISA) using commercially available kits (R&D Systems, Abingdon, Oxon, UK), according to the manufacturer's instructions. Briefly, 10^6^ cells in 100 μl of medium were seeded onto transwell membrane of the inner chamber. After 72 h, 100 μl of supernatants were harvested for ELISA assay.

### Cell transfection

Transfections were performed using Lipofectamine™ 2000 (Ambion) according to the manufacturer's instructions. mTOR small interfering RNA (siRNA) (Ambion) was used to knock down mTOR expression in MSCs. siRNA was mixed with Lipofectamine transfection reagent in serum-free medium according to the manufacturer's instructions. Subsequently, MSCs cultured in 6-well plates to a confluence of 60% were transfected with siRNA in culture medium. The cells were cultivated for a further 48 h at 37°C.

### Statistical analysis

All data were shown as the mean±standard deviation (SD) of at least three independent experiments. All statistical analyses were performed using SPSS 11.0 software, and were analyzed by ANOVA followed by post-hoc Bonferroni tests. *P* < 0.05 was considered to indicate statistically significance.
